# Early Changes in Quantitative Ultrasound Imaging Parameters during Neoadjuvant Chemotherapy to Predict Recurrence in Patients with Locally Advanced Breast Cancer

**DOI:** 10.3390/cancers14051247

**Published:** 2022-02-28

**Authors:** Divya Bhardwaj, Archya Dasgupta, Daniel DiCenzo, Stephen Brade, Kashuf Fatima, Karina Quiaoit, Maureen Trudeau, Sonal Gandhi, Andrea Eisen, Frances Wright, Nicole Look-Hong, Belinda Curpen, Lakshmanan Sannachi, Gregory J. Czarnota

**Affiliations:** 1 Physical Sciences, Sunnybrook Research Institute, Toronto, ON M4N 3M5, Canada; divya.bhardwaj@sri.utoronto.ca (D.B.); archya.dasgupta@sunnybrook.ca (A.D.); daniel.dicenzo@sunnybrook.ca (D.D.); stephen.brade@sunnybrook.ca (S.B.); kashuf.fatima1@sunnybrook.ca (K.F.); karina.quiaoit@sunnybrook.ca (K.Q.); lakshmanan.sannachi@sunnybrook.ca (L.S.); 2 Department of Radiation Oncology, Sunnybrook Health Sciences Centre, Toronto, ON M4N 3M5, Canada; 3 Department of Radiation Oncology, University of Toronto, Toronto, ON M4N 3M5, Canada; 4 Department of Medical Oncology, Department of Medicine, Sunnybrook Health Sciences Centre, Toronto, ON M4N 3M5, Canada; maureen.trudeau@sunnybrook.ca (M.T.); sonal.gandhi@sunnybrook.ca (S.G.); andrea.eisen@sunnybrook.ca (A.E.); 5 Department of Medicine, University of Toronto, Toronto, ON M4N 3M5, Canada; 6 Department of Surgical Oncology, Department of Surgery, Sunnybrook Health Sciences Centre, Toronto, ON M4N 3M5, Canada; frances.wright@sunnybrook.ca (F.W.); nicole.lookhong@sunnybrook.ca (N.L.-H.); 7 Department of Surgery, University of Toronto, Toronto, ON M4N 3M5, Canada; 8 Department of Medical Imaging, Sunnybrook Health Sciences Centre, Toronto, ON M4N 3M5, Canada; belinda.curpen@sunnybrook.ca; 9 Department of Medical Imaging, University of Toronto, Toronto, ON M4N 3M5, Canada; 10 Department of Medical Biophysics, University of Toronto, Toronto, ON M4N 3M5, Canada

**Keywords:** radiomics, breast cancer, recurrence, quantitative ultrasound, neoadjuvant chemotherapy, delta radiomics, machine learning, texture analysis, texture derivatives, imaging biomarker

## Abstract

**Simple Summary:**

Patients diagnosed with breast cancer treated with chemotherapy before surgery were included in this study. The tumor was imaged using ultrasound before the chemotherapy was started and in the middle of chemotherapy treatment (one month after starting). After treatment completion, patients were followed up according to standard clinical practice and categorized into two groups based on disease recurrence until the last follow-up. The ultrasound imaging was analyzed using advanced computational techniques, and artificial intelligence was used to develop models to differentiate between the two outcomes. We demonstrated that using the ultrasound image data, the final outcomes can be predicted as early as within one month of the start of chemotherapy.

**Abstract:**

Background: This study was conducted to explore the use of quantitative ultrasound (QUS) in predicting recurrence for patients with locally advanced breast cancer (LABC) early during neoadjuvant chemotherapy (NAC). Methods: Eighty-three patients with LABC were scanned with 7 MHz ultrasound before starting NAC (week 0) and during treatment (week 4). Spectral parametric maps were generated corresponding to tumor volume. Twenty-four textural features (QUS-Tex^1^) were determined from parametric maps acquired using grey-level co-occurrence matrices (GLCM) for each patient, which were further processed to generate 64 texture derivatives (QUS-Tex^1^-Tex^2^), leading to a total of 95 features from each time point. Analysis was carried out on week 4 data and compared to baseline (week 0) data. ∆Week 4 data was obtained from the difference in QUS parameters, texture features (QUS-Tex^1^), and texture derivatives (QUS-Tex^1^-Tex^2^) of week 4 data and week 0 data. Patients were divided into two groups: recurrence and non-recurrence. Machine learning algorithms using *k*-nearest neighbor (k-NN) and support vector machines (SVMs) were used to generate radiomic models. Internal validation was undertaken using leave-one patient out cross-validation method. Results: With a median follow up of 69 months (range 7–118 months), 28 patients had disease recurrence. The k-NN classifier was the best performing algorithm at week 4 with sensitivity, specificity, accuracy, and area under curve (AUC) of 87%, 75%, 81%, and 0.83, respectively. The inclusion of texture derivatives (QUS-Tex^1^-Tex^2^) in week 4 QUS data analysis led to the improvement of the classifier performances. The AUC increased from 0.70 (0.59 to 0.79, 95% confidence interval) without texture derivatives to 0.83 (0.73 to 0.92) with texture derivatives. The most relevant features separating the two groups were higher-order texture derivatives obtained from scatterer diameter and acoustic concentration-related parametric images. Conclusions: This is the first study highlighting the utility of QUS radiomics in the prediction of recurrence during the treatment of LABC. It reflects that the ongoing treatment-related changes can predict clinical outcomes with higher accuracy as compared to pretreatment features alone.

## 1. Introduction

Breast cancer is the most commonly diagnosed malignancy and is the leading cause of cancer-related death among women [1]. Locally advanced breast cancer (LABC) can be clinically defined as a primary tumor larger than 5 cm in size, which may be fixed to the chest wall, with advanced lymph node involvement. LABC poses a clinical challenge because, despite aggressive multimodality treatment, these patients have a high rate of recurrence [2,3]. Neoadjuvant chemotherapy (NAC) is often used in patients with LABC with the purpose of downstaging disease, improving resectability, and also treatment response can serve as a useful indicator of long-term survival outcomes in specific molecular subgroups [4]. Approximately 30–50% of patients with LABC after primary treatment present with recurrence, which is influenced by prognostic factors as well as treatment strategies [2]. For breast cancer patients, it has been demonstrated that the fear of cancer recurrence is one of the most common and aversive psychological phenomena [5]. Genomic tools have been developed in estimating recurrence risk in patients with early-stage breast cancer and incorporated into clinical practice, guiding decisions regarding adjuvant therapy [6,7,8]. However, most of these genomic assays have limited clinical utility in LABC so far. In regards to imaging tools, limited literature exists on the use of imaging biomarkers in predicting the risk of breast cancer recurrence. In the past decade, a growing interest in the field of radiomics using quantitative imaging analysis has led to significant advancements in image-based phenotyping aimed at tumor characterization, and recently the identification of predictors of treatment response in patients with breast cancer [9]. Radiomics reflects the mechanisms occurring at a genetic and molecular level by converting imaging data into high dimensional, mineable quantitative features.

Ultrasound (US) is a commonly used imaging modality with widespread clinical applications in differentiating benign versus malignant primary lesions in the breast or characterizing lymph nodes and facilitating tissue acquisition for histological evaluation. Quantitative ultrasound (QUS) is similar to the clinically used ultrasound device, with the primary difference of acquisition and analyzing the raw radiofrequency (RF) data, which aids in more detailed biological characterization of the imaged tissues. The biophysical basis of QUS imaging is the differences in the elastic properties, leading to differential scattering reflected as backscatter ultrasound signals from tissues [10,11]. Application of quantitative texture analysis to the QUS imaging has shown to unfold critical biological information that can be modeled effectively to predict biological behavior in breast malignancies [12,13]. Several studies have demonstrated the utility of QUS imaging in predicting treatment response using pretreatment imaging features [12,14,15,16]. Similarly, the acquisition of QUS features during different treatment modalities (chemotherapy, radiotherapy) reflects ongoing microstructural changes resulting from cell death and can effectively improve the interpretation of clinical outcomes [17,18].

The study here explored the role of QUS features, including higher-order texture features obtained during the early course of NAC, in predicting the recurrence for patients with LABC. We have demonstrated the utility of QUS in predicting clinical outcomes with reasonable accuracy. Being a portable technique with rapid scan acquisition, QUS may be beneficial over other imaging modalities for response monitoring mid-treatment as well as in characterizing long term outcomes.

## 2. Material and Methods

### 2.1. Patient Selection

The prospective study was approved by the Research Ethics Board at Sunnybrook Health Sciences Centre (SHSC), Toronto, Canada, and registered with Clinical Trials.gov (NCT00437879). All study participants signed a written consent form. Inclusion criteria were the presence of biopsy-proven LABC and scheduled to receive NAC as part of their standard care of treatment. Clinical information was obtained from a prospectively maintained database with additional information collected from patient electronic medical records. Patients diagnosed with inflammatory breast cancer or upfront metastasis were excluded from the analysis. The time to recurrence was defined from the start of NAC to the clinical or radiological evidence of disease recurrence. In order to avoid underestimating the risk of recurrence, the minimum follow-up time for patients without any recurrence was required to be at least four years.

### 2.2. Treatment Details

The majority of patients were treated with an anthracycline and taxane-based NAC, while some patients received other chemotherapy treatments. The chemotherapy regimen and the interval between two cycles were based on the decision of the treating medical oncologist. Patients with human epidermal growth factor receptor 2 (HER2) positive status received targeted therapy in combination with NAC. After receiving NAC, all patients underwent surgery (mastectomy or breast-conserving surgery), in conjunction with sentinel lymph node biopsy (SLNB) or axillary lymph node dissection (ALND), based on the pretreatment disease stage and response. Patients received adjuvant locoregional radiation therapy and maintenance targeted therapy or endocrinal therapy according to standard institutional practice. Patients were followed up every 3–6 months during the first two years, and then after every 6–12 months, as indicated clinically. Oncologists confirmed all recurrences with clinical examination, diagnostic imaging, and histological confirmation as indicated clinically.

### 2.3. Quantitative Ultrasound Parameter Estimation

Ultrasound data were obtained using a Sonix RP clinical research system (Analogic Medical Corp., Vancouver, Canada) coupled to a L14–5/60 transducer with a central frequency of 7 MHz and −6 dB bandwidth of 4–9 MHz. Data were digitally collected with a sampling frequency of 40 MHz with a 16-bit resolution. Patients were scanned before the initiation of systemic therapy (week 0) and at week 4 from the start date of NAC. The scan frames were collected at intervals of 1 cm, spanning the entire tumor, with the transducer focus towards the center of the tumor. On each ultrasound frame, a region of interest (ROI) was manually contoured corresponding to the tumor. Using a sliding window approach, each ROI was divided into analysis blocks of size 10λ × 10λ, with a 94% adjacent overlap in axial and lateral directions (2.2 mm × 2.2 mm approximately). The detailed description of standardization methods and calculation of quantitative ultrasound parameters have been explained in previous publications [19]. In short, from each of the selected ultrasound frames, spectral parameters including mid-band fit (MBF), spectral slope (SS), spectral intercept (SI), spacing among scatterers (SAS) and backscatter model parameters, acoustic scatterer diameter (ASD) Anderson, and average acoustic-scatterer concentration (AAC) were determined [20,21]. Attenuation coefficient estimation (ACE) was used as a spectral correction factor and also as a predictive parameter [22,23]. Spectral parametric maps, represented as color-coded image maps, were generated for each ROI through the spatial mapping of the parametric values calculated from all window blocks. Mean QUS parameters were calculated by averaging the values within the maps. The workflow for feature extraction, normalization process has been presented in Supplementary Figure S1.

### 2.4. Texture Features and Texture Derivatives Evaluation

The spatial distribution of QUS parameters was evaluated from parametric maps using grey-level co-occurrence matrices (GLCM) [24], representing the distance and angular spatial relationship between neighboring pixels. From each parametric map, the following four textural features were extracted: Contrast parameter (CON), correlation parameter (COR), homogeneity parameter (HOM), and energy parameter (ENE).

In summary, the CON parameter measures the magnitude of intensity differences between two neighboring pixels. The COR parameter is a measure of how linearly a pixel is correlated to its adjacent pixel. The ENE parameter measures textural uniformity, and the HOM parameter estimates the incidence of pixel pairs of different intensities. A total of 24 textural features (QUS-Tex^1^) were determined from the six spectral parametric maps (excluding ACE).

In order to determine third-order imaging features (texture derivatives), the GLCM method (ENE, CON, COR, HOM) was repeated on the 16 QUS-texture maps (excluding SAS and SS textural features) described above. As a result, a total of 64 texture derivatives were determined (QUS-Tex^1^-Tex^2^). The steps were repeated for all ultrasound scans obtained (at weeks 0 and 4). The ∆week 4 data was obtained from the difference in mean QUS parameters, texture features (QUS-Tex^1^), and texture derivatives (QUS-Tex^1^-Tex^2^) of week 4 data from the corresponding week 0 data.

Therefore we had a set of 95 features from each time point (seven QUS spectral features, 24 QUS-Tex^1^, and 64 QUS Tex^1^-Tex^2^). The list of all the features extracted is presented in Supplementary File. Final analysis was carried out using a combination of week 0 and ∆week 4 data (190 features). Image processing, feature extraction, development of parametric maps, and texture analysis were done using MATLAB 2016a software.

### 2.5. Statistical Analysis and Classification Algorithms

A Shapiro–Wilk normality test was applied to the feature sets to confirm data distribution for the two groups (patients with and without recurrence). For normally distributed faculty, a parametric unpaired student *t*-test was used, and otherwise, a non-parametric test Mann–Whitney U test was performed. A total of 190 features (7 mean QUS, 24 QUS-Tex^1^, 64 QUS-Tex^1^-Tex^2^ from each point), which was a combination of week 0 data and ∆Week 4 data, were used for the model development. A *p*-value of < 0.05 was considered statistically significant.

Two machine learning classifiers, consisting of k-nearest neighbor (k-NN), and support vector machine-radial based function (SVM), were independently tested for comparison using MATLAB 2016a to evaluate diagnostic performance. The maximum feature number was set to 3 in the classification model to avoid model overfitting due to the high dimensionality of the data set [25]. Since the two groups were not balanced well (28 with recurrence and 55 without recurrence), a subset sampling method was undertaken. Seven subsets were randomly generated to select an equal number of patients equivalent to the smaller group (recurrence), with the final class assignment done based on majority voting. A forward feature selection method was used for building the classifier algorithms. A leave one patient out cross-validation method was used, which uses all except one data point in the prediction of the output class. The performances of the different algorithms were compared based on different diagnostic indices obtained, including sensitivity, specificity, accuracy, and area under curve (AUC) along with 95% confidence intervals. To test the incremental values of higher-order features (texture derivatives), the classifier models were tested separately including QUS and QUS-Tex1 features, and then with all the features combined.

Survival analysis was undertaken using the Kaplan–Meier product-limit method. Univariate analysis was done using log-rank tests to compare the survival estimates for the two predicted groups using the radiomics model (predicted recurrence group versus predicted non-recurrence group).

## 3. Results

### 3.1. Clinical Characteristics

The analysis included a sample size of 83 women with LABC as per inclusion criteria. The patient’s ages ranged from 29 to 79 years (median 50 years). Clinical characteristics, histopathologic features, and treatment details are mentioned in Table 1. To summarize, the histology was invasive ductal carcinoma (IDC) in 76, invasive lobular carcinoma (ILC) in 3, mixed IDC and ILC in 3, and metaplastic carcinoma in 1. Fifty-six patients received doxorubicin, cyclophosphamide, and paclitaxel (AC-T), and twenty patients were administered fluorouracil, epirubicin, cyclophosphamide, and docetaxel (FEC-D). Twenty-nine patients were HER2 positive and, forty-eight were estrogen receptor (ER) positive.

### 3.2. Quantitative Ultrasound Feature Analysis

**Figure 1:**
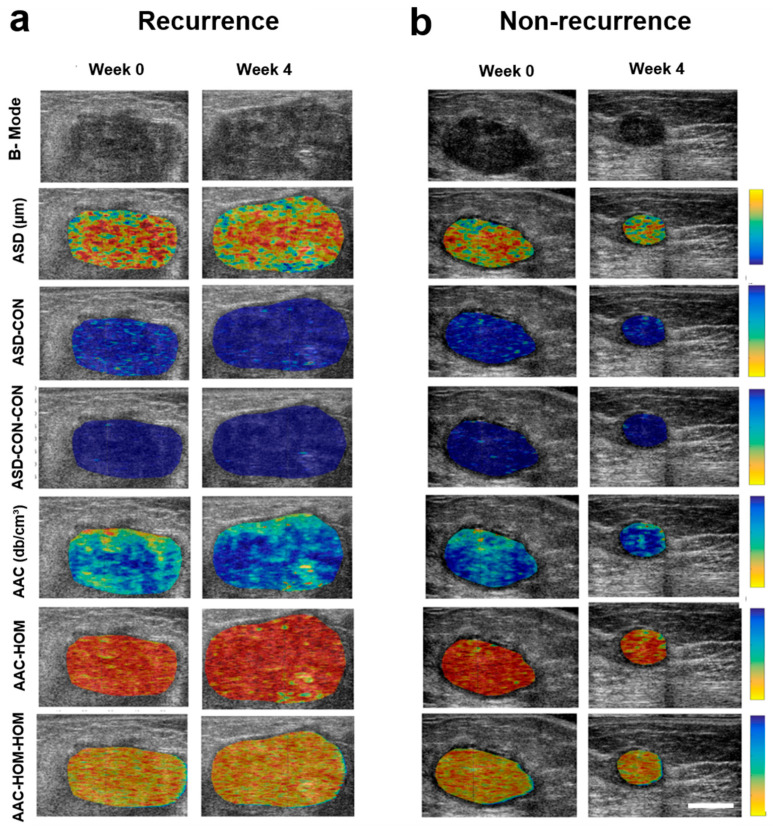
Quantitative ultrasound parametric images for representative patients with and without disease recurrence. Representative ultrasound B-mode images and QUS-derived parametric maps (ASD, ASD-CON, ASD-CON-CON, AAC, AAC-HOM, AAC-HOM-HOM) from one patient each with recurrence (**a**) and no recurrence (**b**) acquired at week 0 and week 4 of treatment. The color maps represent the quantitative values of the spectral parameters within the tumor. The change in values of the parameters with treatment can be appreciated by the change of assigned color to the sub-regions of interest within the tumor. The color scale on the right side represents the range for individual features, ASD parameter of 40 to 200 µm, ASD-CON texture feature of 0 to 20, ASD-CON-CON texture derivative of 0 to 54, AAC parameter of 7 to 65 db/cm^3^, AAC-HOM texture feature of 0 to 1, AAC-HOM-HOM texture derivative of 0 to 1. The scale bar represents 2 cm.

**Figure 2:**
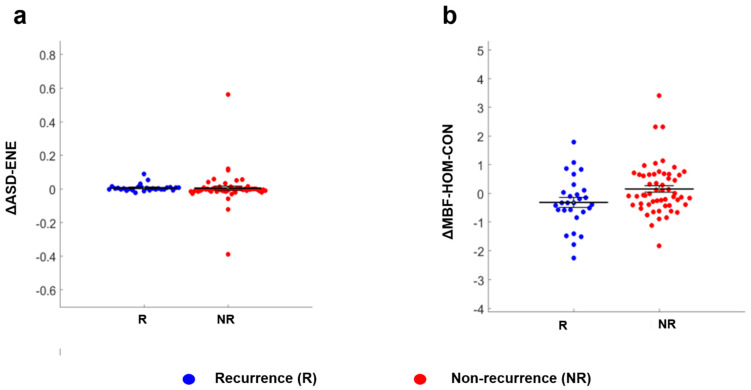
Scatter plots for the features having significantly different values for patients with and without recurrence. Legends: Figure 2 shows the distribution of values from all patients represented in the recurrence (blue circle) and without recurrence (red circle) groups for ∆ASD-ENE (**a**) and ∆MBF-HOM-CON (**b**). ∆ Indicates the difference of values of week 4 from week 0 for each feature included in the analysis.

Representative ultrasound B-mode images and parametric maps of QUS features and selected texture and texture-derivative maps for a patient with (1a) and without recurrence (1b) are presented in Figure 1. ∆ASD-ENE (*p* = 0.033), and ∆MBF-HOM-CON (*p* = 0.038) parameters exhibited a statistically significant difference between the two groups of patients (Table 2). The corresponding scatter plots for these two features as obtained from all the patients are shown in Figure 2.

### 3.3. Classifier Results

**Figure 3:**
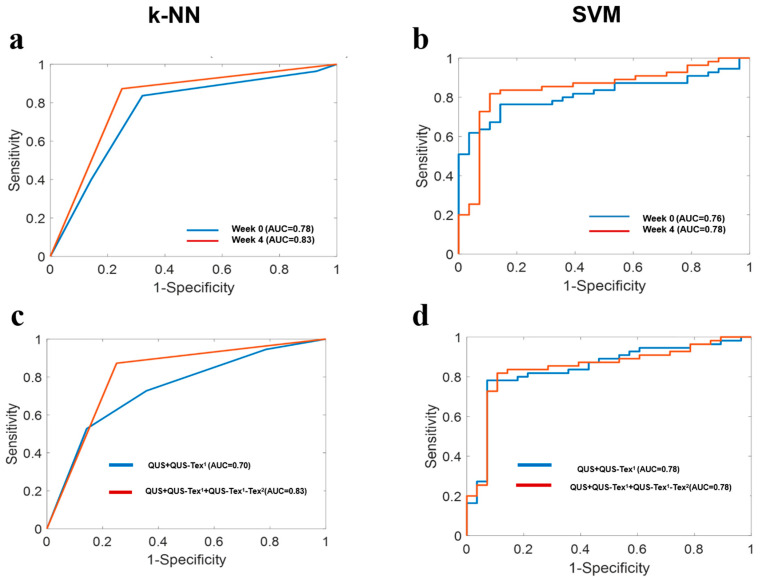
Receiver operating characteristic (ROC) plots showing the estimated area under curve (AUC) values obtained from the two classifiers. (**a**,**b**) A comparison of the performance by the two classifiers (k-NN and SVM) based on week 0 and week 4 time-points using all features. (**c**,**d**) The comparison of the performance of QUS + QUS-Tex^1^ (without texture derivatives) and QUS + QUS-Tex^1^ + QUS-Tex^1^-Tex^2^ (with texture derivatives) data set by k-NN and SVM models at week 4.

Results obtained using different classifier models are displayed in Table 3, and their associated receiver operating characteristic (ROC) curves and AUC values are presented in Figure 3. The performance of both classifier models, k-NN and SVM based on data acquired at a week 4 time, had an accuracy above 70%. Specifically, it was observed that using the k-NN classifier model, the week 4 data in aggregate (QUS + QUS-Tex^1^ + QUS-Tex^1^-Tex^2^) performed better as compared to the week 0 data (QUS + QUS-Tex^1^ + QUS-Tex^1^-Tex^2^). The AUC improved from 0.78 to 0.83 in the k-NN classifier model, whereas it only enhanced marginally in the SVM model, from 0.76 to 0.78 (Figure 3a,b).

At week 4, there was a significant improvement observed in the performance of the k-NN classifier model with the introduction of texture derivatives (QUS-Tex^1^-Tex^2^). The AUC improved significantly from 0.70 to 0.83 (Figure 3c). For the SVM model, the AUC of 0.78 remained the same, with no difference seen with the incorporation of texture derivatives (Figure 3d).

Overall, the k-NN classifier performed best at week 4; the selected best features were a multiparametric combination of ACE_W0_, AAC-CON-CON_W0_, and ∆ASD-CON-CON. The sensitivity, specificity, and accuracy at week 4 for this model were 87%, 75%, and 81%, respectively.

### 3.4. Clinical Outcomes and Performance of Prediction Models

**Figure 4:**
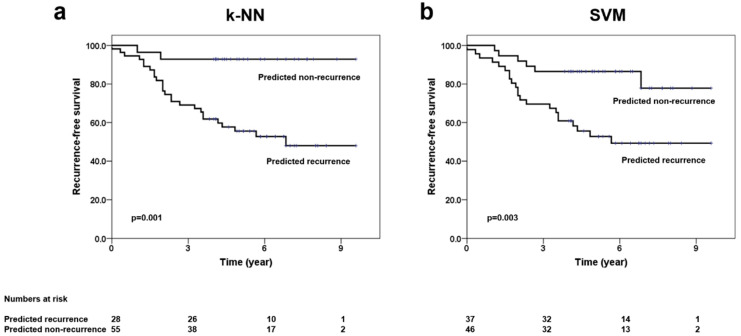
Kaplan–Meier survival plots showing recurrence-free survival based on predicted groups (recurrence vs. no recurrence). Legends: Survival curves showing the differences in the recurrence-free survival outcomes as obtained from the predicted groups using the k-NN model (**a**) and SVM model (**b**) at week 4 time-point, including all features (QUS + QUS-Tex^1^ + QUS-Tex^1^-Tex^2^).

The median follow up for the entire cohort was 69 months (interquartile range 49–84 months). Out of eighty-three patients included in the analysis, 28 patients had developed disease recurrence, with the median time to recurrence being 24 months. More than 80% of the recurrences were seen in the first four years. The site of the first recurrence was isolated local disease in five, local and regional in one, regional and distant in four, isolated distant relapse in 17, and combined local, regional, and distant in one patient. For the entire group, five-year recurrence-free survival (RFS) and five-year overall survival (OS) were 68% and 79%, respectively. The predicted RFS using the k-NN and SVM classifier is shown in Figure 4. The five-year RFS for the predicted recurrence and non-recurrence groups using the k-NN classifier was 56% (95% confidence interval 44–69%) and 93% (95% confidence interval 84–99%), respectively (*p* = 0.001), and presented in Supplementary Table S1.

## 4. Discussion

*Statement of principal findings*: In the study, 83 patients with LABC were imaged using QUS before starting NAC and at week 4 of treatment. The changes in spectral parameters, texture and higher-order texture-derivate features from chemotherapy were computed and radiomics models were developed with disease recurrence as the endpoint. A k-NN classifier was able to predict recurrence with sensitivity, specificity, accuracy, and AUC of 87%, 75%, 81%, and 0.83, respectively.

*Strengths and weakness*: This is the first study demonstrating the ability of QUS obtained during the course of NAC in predicting disease recurrence in patients with breast cancer. The study included patients with diverse clinical and molecular characteristics suggesting the applicability of QUS radiomics model across different populations of patients with breast cancer. We had a reasonable follow up (median follow up > 5 years), with clinical outcomes recorded prospectively adding to the strengths of the study. One of the limitations of the study here was the relatively small number of patients who presented with recurrence. The inclusion of more patients will likely improve the performance and robustness of the classifiers, and also extending the study at other centers will help in undertaking external validation techniques.

*Relation to other studies:* Several studies have identified various factors that can predict the recurrence of breast cancer ranging from clinical characteristics, immunohistochemical assays to gene expression levels. Clinically, the most reliable prognostic markers include nodal status and tumor size. Various other clinical factors such as tumor grade, patient age, and treatment type have been added to build a Clinical Treatment Score (CTS), which provides a recurrence risk estimate for ER+ breast cancer [26]. In the past two decades, there has been a range of genetic markers to predict the risk of cancer recurrence, primarily for EBC [6,27,28]. Some studies have compared the genetic analysis with imaging features showing good correlations. In a study by Sutton et al. (2015), the association between the gene assay recurrence score and texture-based image features extracted from magnetic resonance imaging (MRI) was investigated [29]. Similarly, a study by Woodard et al. (2018) showed equivalent efficacy of the gene assay and Breast Imaging and Reporting Data System (BI-RADS) mammography and MR images in the prediction of recurrence [30].

*Meaning of the study*: The role of ultrasound is widely recognized in the screening and diagnosis of breast masses. Applications include assessing morphological tumor details (spiculated, rounded, with necrosis, microcalcification), anatomic relationships of masses to their surrounding tissues, and regional lymph nodal involvement. However, recent clinical applications of ultrasound have expanded in the management of breast cancer. For example, ultrasound elastography techniques can be used to differentiate benign from malignant breast lesions, contrast-enhanced ultrasound can characterize tissues with different vascularity, and three-dimensional ultrasound improves the characterization of breast lesions [31]. QUS Techniques have been applied in characterizing tumor masses and predicting treatment response in patients with LABC [17,32].

In the past, it has been demonstrated in preclinical studies that QUS can evaluate cell death in response to different treatment modalities [33,34]. The intracellular mechanisms resulting in phenotypical cell changes differ according to the mode of cell death. For example, mitotic arrest results in cell swelling, whereas apoptotic death leads to cell shrinkage, chromatin condensation, and nuclear fragmentation. These events form the basis for differential change in QUS spectral and textural parameters for cell death monitoring. These observations have been interpreted in clinical studies to predict treatment response within 4 weeks of initiation of NAC [35].

The work presented in this study aimed to investigate the effectiveness of QUS texture derivatives in predicting recurrence risk rather than the local response in breast cancer within weeks of initiation of systemic therapy. The improvement in AUC performance with the addition of texture derivatives, as well as from week 0 to week 4 in the k-NN classifier [36], signifies the importance of higher-order derivatives and continuous monitoring. The improvement in classifier performance with inclusion of week 4 parameters suggests the ongoing changes in the elastic properties of the tumor induced by chemotherapy and its reflection on dictating the biological behaviour. It was interesting to observe that the best features chosen by the k-NN model to classify patients in the two groups mainly were texture derivatives (texture of texture features). The features were a combination of ACE_W0_, AAC-CON-CON_W0_, and ∆ASD-CON-CON, at week 4 (Week 0 + ∆Week 4). Amongst the three features, the two parameters (ACE_W0_ & AAC-CON-CON_W0_) are related to tissue composition and microstructural organization of cells. The selection of texture derivative ‘AAC-CON-CON_W0′_ technically represents the summation of other sub-variables and possibly depicts heterogeneity within a tumor at a more advanced level. Other parameters were selected from the pretreatment (week 0) data. This suggests that the spatial organization of the tissue as reflected in QUS parameters relates to tumor biology and may have a role in recurrence prediction to some extent before initiation of treatment. In previous studies, ACE _W0_ was found to be a significant predictive parameter to distinguish tissues of different types, supporting the findings in this study [37,38].

The other texture derivative selected was ∆ASD-CON-CON, where ASD signifies microstructural size and may be related to lobular diameter, and the contrast derivatives (contrast of contrast), which measure the intensity differences. This was found to be significant after the initiation of treatment. As mentioned previously, changes occurring at the molecular level in tumor cells in response to the treatment can be captured by the QUS technique. Hence, it is postulated that, as treatment initiates, ASD and its derivatives have a potential role in predicting recurrence risk and differentiating the two groups.

We estimated the survival outcomes based on a prediction by the two machine learning classifiers. Out of the two, the k-NN classifier using texture derivatives (week 4) was able to closely approximate the curve obtained from the patient’s clinical details on recurrence, thus displaying the efficacy of the classifier in recurrence prediction. Magnetic resonance imaging and mammography are used more commonly in clinical practice for imaging of breast malignancies aiding in diagnosis, staging, and response evaluation to treatment. Ultrasound forms an attractive strategy due to its portable nature, ability of rapid scan acquisition, no radiation hazards, and better patient compliance. Quantitative analysis of the ultrasound imaging also provides an opportunity to capture the changes in the tumor in real-time during treatment, much earlier than other imaging modalities which rely on the morphological changes.

*Unanswered question and future research:* In the future, with a larger cohort, it may also be worthwhile to combine clinical features and molecular subgroups with a QUS-radiomics model. The promising results obtained in the study here emphasize the importance of QUS parameters as a valuable tool for the timely identification of patients whose tumors have a strong tendency towards recurrence. An early prediction of recurrence risk can potentially assist oncologists in making decisions in regards to selecting systemic agents for treatment or changing a less effective treatment to more effective therapy or maintenance therapies. It may provide an insight into an earlier shift to surgery or towards an intensification of systemic therapy before missing the ‘therapeutic window’ for benefit.

## 5. Conclusions

To summarize, this study demonstrates the effectiveness of QUS-radiomics in predicting recurrence in patients with LABC early in the course of treatment. The work presented here highlights the role of higher-order imaging features (texture derivatives) in improving the performance of the classifiers at four weeks into neoadjuvant chemotherapy. Further studies externally validating these initial, single-center findings are warranted.

## Figures and Tables

**Table 1:** Patient, disease, and treatment-related characteristics for all patients (*n* = 83).

Features		Recurrence (*n* = 28)		Non-Recurrence (*n* = 55)	
Patient Characteristics		*n*	%	*n*	%
Age	Median (Range)	50 (29–79) years		48 (31–72) years	
Menopausal Status	Premenopausal	16	57	33	60
	Perimenopausal	1	4	3	6
	Postmenopausal	10	36	17	31
	Not specified	1	4	2	4
Laterality	Right	15	54	27	49
	Left	13	46	28	51
**Pathological features**		***n***	**%**	***n***	**%**
Histology	IDC	25	89	51	93
	ILC	2	7	1	2
	Others	1	4	3	5
HR+/HER2+		6	21	14	26
HR+/Her2−		10	36	20	36
HR−/HER2+		4	14	5	9
TNBC		8	29	16	29
**Neoadjuvant Treatment**		***n***	**%**	***n***	**%**
Chemotherapy regimen	AC-T	21	75	35	64
	FEC-D	5	18	15	27
	TC	2	7	5	8
Dose Dense	No	13	46	26	47
	Yes	15	54	29	53
Trastuzumab	No	18	64	36	66
	Yes	10	36	19	34
**Treatment Response**		***n***	**%**	***n***	**%**
Pathological Complete Response (pCR)		0	0	16	29
Partial Responder (PR)		21	75	33	60
Non Responder (NR)		7	25	6	11

Abbreviations: HR: Hormone receptor; HER2+: Human epidermal growth factor receptor 2; IDC: Invasive ductal carcinoma; ILC: Invasive lobular carcinoma; AC-T: doxorubicin, cyclophosphamide, and docetaxel, FEC-D: 5-fluorouracil, epirubicin, cyclophosphamide, and docetaxel; TC: docetaxel and cyclophosphamide.

**Table 2:** Features with significant differences at week four into neoadjuvant chemotherapy.

Parameter	Recurrence Mean ± SEM	Non-Recurrence Mean ± SEM	*p*-Value
∆ASD-ENE	0.008 ± 0.021	0.005 ± 0.099	0.033
∆MBF-HOM-CON	−0.306 ± 0.889	0.160 ± 0.867	0.038

∆ Indicates the difference of values of week 4 from week 0 for each feature included in the analysis. Abbreviations: SEM: standard error of the mean; R: Recurrence; NR: No Recurrence; ASD: Average Scatterer Diameter; MBF: Mid-band fit; AAC: Average Acoustic Concentration; ENE: Energy; HOM: Homogeneity; CON: Contrast.

**Table 3:** Classification performance of the two machine learning classifiers with the selected features.

Classification Performance	Model	%Sn	%S_p_	%Acc	AUC	Selected Feature(s)
**First and second-order** **(QUS + QUS-Tex^1^)**	k-NN	73 (61–83)	64 (52–75)	74 (63–85)	0.70 (0.59–0.79)	ΔSAS ΔASD-ENE ASD-CON_W0_
	SVM	74 (64–83)	86 (77–95)	84 (72–95)	0.78 (0.66–0.89)	SAS_W0_ ASD-CON_W0_ ΔAAC-HOM
**All features** **(QUS + QUS-Tex^1^ +** **QUS-Tex^1^-Tex^2^)**	**k-NN**	**87** **(78–95)**	**75** **(64–85)**	**81** **(70–93)**	**0.83** **(0.73–0.92)**	**ACE_W0_** **AAC-CON-CON_W0_** **ΔASD-CON-CON**
	SVM	75 (62–87)	85 (72–96)	85 (73–97)	0.78 (0.68–0.88)	SAS_W0_ ASD-CON_W0_ ΔAAC-HOM

∆ Indicates the difference of values of week 4 from week 0 for each feature included in the analysis. The best classifier performances using the k-NN model have been highlighted in bold. The values in parenthesis represent 95% confidence interval. Abbreviations: S_n_: Sensitivity; S_p_: Specificity, Acc: Accuracy, AUC: Area under curve; *k-*NN: *k*-nearest-neighbors; SVM: Support vector machine with radial based kernel function; AAC (dB/cm^3^): Average Acoustic Concentration; ASD (µm): Average Scatterer Diameter; SAS: Spacing Among Scatterer; ACE (dB/cm-MHz): Attenuation Coefficient Estimate; CON: Contrast; HOM: Homogeneity; ENE: Energy.

## Data Availability

Anonymized data will be provided on request to the corresponding author according to institutional ethics committee guidelines.

## Supplementary Materials

The following are available online at https://www.mdpi.com/article/10.3390/cancers14051247/s1, Figure S1: Study methodology summarizing feature processing, Table S1: The survival outcomes (recurrence-free survival) as predicted by the classifiers after the development of the model. Supplementary File: List of all imaging features.
