# Supplementary material for: Early Changes in Quantitative Ultrasound Imaging Parameters during Neoadjuvant Chemotherapy to Predict Recurrence in Patients with Locally Advanced Breast Cancer
Source: Cancers (Basel). 2022 Feb 28;14(5):1247. doi: 10.3390/cancers14051247 (PMC8909335; doi:10.3390/cancers14051247)
Supplement: Supplementary file 1 [file cancers-14-01247-s001.zip › Supplementary File.pdf]

Spectral features

MBF

SS

SI

SAS

ACE

ASD

AAC

Texture features

MBF_CON

MBF_COR

AAC_CON_CON

AAC_CON_COR

AAC_CON_ENE

AAC_CON_HOM

AAC_COR_CON

AAC_COR_COR

AAC_COR_ENE

AAC_COR_HOM

AAC_ENE_CON

AAC_ENE_COR

AAC_ENE_ENE

AAC_ENE_HOM

AAC_HOM_CON

AAC_HOM_COR

AAC_HOM_ENE

AAC_HOM_HOM

ASD_CON_CON

MBF_ENE

MBF_HOM

SS_CON

SS_COR

SS_ENE

SS_HOM

SI_CON

SI_COR

SI_ENE

SI_HOM

SAS_CON

SAS_COR

ASD_CON_COR

ASD_CON_ENE

ASD_CON_HOM

ASD_COR_CON

ASD_COR_COR

ASD_COR_ENE

ASD_COR_HOM

ASD_ENE_CON

ASD_ENE_COR

ASD_ENE_ENE

ASD_ENE_HOM

ASD_HOM_CON

ASD_HOM_COR

ASD_HOM_ENE

ASD_HOM_HOM

MBF_CON_CON

MBF_CON_COR

SAS_ENE

SAS_HOM

ASD_CON

ASD_COR

ASD_ENE

ASD_HOM

AAC_CON

AAC_COR

AAC_ENE

AAC_HOM

Texture derivatives

MBF_CON_ENE

MBF_CON_HOM

MBF_COR_CON

MBF_COR_COR

MBF_COR_ENE

MBF_COR_HOM

MBF_ENE_CON

MBF_ENE_COR

MBF_ENE_ENE

MBF_ENE_HOM

MBF_HOM_CON

MBF_HOM_COR

MBF_HOM_ENE

MBF_HOM_HOM

SI_CON_CON

SI_CON_COR

SI_CON_ENE

SI_CON_HOM	SI_ENE_CON	SI_HOM_COR
SI_COR_CON	SI_ENE_COR	SI_HOM_ENE
SI_COR_COR	SI_ENE_ENE	SI_HOM_HOM
SI_COR_ENE	SI_ENE_HOM	
SI_COR_HOM	SI_HOM_CON	

MBF: Mid-band fit, SS: Spectral slope, SI: Spectral intercept, SAS: Spacing among scatterers, ACE: Attenuation coefficient estimation, ASD: Acoustic scatterer diameter (ASD)- Anderson and average acoustic-scatterer concentration (AAC), CON: Contrast, COR: Correlation parameter, HOM: Homogeneity, ENE: Energy
