# Supplementary material for: Early Changes in Quantitative Ultrasound Imaging Parameters during Neoadjuvant Chemotherapy to Predict Recurrence in Patients with Locally Advanced Breast Cancer
Source: Cancers (Basel). 2022 Feb 28;14(5):1247. doi: 10.3390/cancers14051247 (PMC8909335; doi:10.3390/cancers14051247)
Supplement: Supplementary file 1 [file cancers-14-01247-s001.zip › Supplementary.pdf]

Table S1. The survival outcomes (recurrence-free survival) as predicted by the classifiers after the development of the model. Abbreviations: k-NN: k Nearest Neighbors; SVM: Support Vector Machine with radial basis function kernel.

Classifiers	Predicted Group	5-year predicted RFS (95% confidence interval)	*p*-value
k-NN	Recurrence	56% (44–69%)	0.001
	Non-Recurrence	93% (84–99%)	
SVM	Recurrence	53% (38–68%)	0.003
	Non-Recurrence	87% (76–97%)	

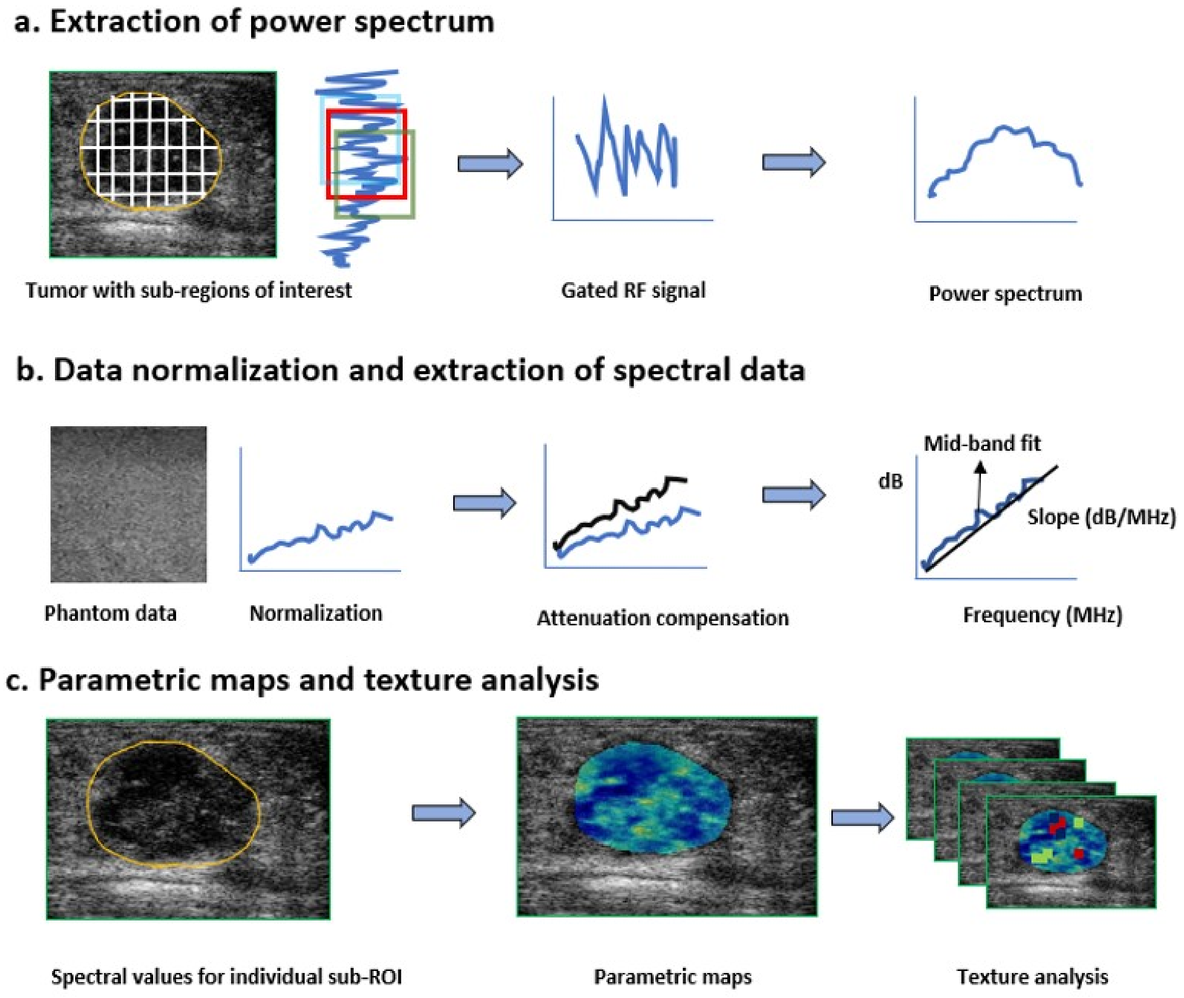

Figure S1. Study methodology summarizing feature processing.
